# Interaction Between PRDM14 and CBFA2T2 Supports Pluripotency and Proliferation in Germ Cell Tumors

**DOI:** 10.3390/cancers18132090

**Published:** 2026-06-27

**Authors:** Deana Leah Wood, Aaron Michael Taylor, Jody Therieault Lombardi, Patrick Kwok Shing Ng, Ching C. Lau, Joanna J. Gell

**Affiliations:** 1 The Jackson Laboratory for Genomic Medicine, Farmington, CT 06032, USA; 2 Department of Pediatrics, School of Medicine, University of Connecticut, Farmington, CT 06032, USA; 3 Center for Cancer and Blood Disorders, Connecticut Children’s, Hartford, CT 06106, USA

**Keywords:** germ cell tumors, pluripotency, proliferation, embryonal carcinoma, siRNA knockdown, RNA-sequencing

## Abstract

**Background/Objectives:** Germ cell tumors (GCTs) are thought to arise from primordial germ cells that fail to appropriately differentiate and instead retain pluripotency programs. PRDM14 is a key regulator of pluripotency and primordial germ cell specification and is aberrantly expressed in multiple GCT subtypes. However, the role of PRDM14 in GCT malignancy remains unclear. In this study, we investigated whether PRDM14 functions in GCTs through CBFA2T2, a transcriptional corepressor previously identified as a PRDM14-interacting partner in pluripotent stem cells and developmental models. **Methods:** To determine the presence and level of PRDM14 and CBFA2T2 in GCT, a panel of GCT lines were assessed for RNA and protein expression and interaction. Then, to better understand the biological effects of PRDM14 and CBFA2T2 within GCTs, *PRDM14* and *CBFA2T2* knockdowns were employed. **Results:** We show that PRDM14 and CBFA2T2 are expressed across GCT cell lines, colocalize predominantly in the nucleus, and cooperate as a complex in GCT cell lines. Knockdown of either *PRDM14* or *CBFA2T2* resulted in reduced expression of key pluripotency genes and a significant impairment of cell proliferation, indicating a shared role in maintaining an undifferentiated, proliferative state. Transcriptomic analysis following *PRDM14* or *CBFA2T2* depletion revealed extensive overlap in differentially expressed genes and convergent alterations in developmental and metabolic signaling pathways. **Conclusions:** Together, these findings suggest that PRDM14 and CBFA2T2 form a functional complex that sustains pluripotency and proliferation in GCT cells. This supports a model in which persistence of germline regulatory mechanisms contributes to GCT malignancy, highlighting this interaction as a novel component of GCT biology.

## Simple Summary

Germ cell tumors (GCTs) are unique tumors affecting children, adolescents and young adults. One defining feature of some types of GCTs is retained stem-cell-like properties. In many cancers, these features represent therapeutic vulnerabilities. Here, we present *PRDM14*, a regulatory gene that may cooperate with *CBFA2T2* to keep cells in a pluripotent, or undifferentiated state. Our work aimed to understand how PRDM14 and CBFA2T2 might influence GCT biology by examining how these genes interact and reducing the amount of each gene to observe how cell health and other genes were affected in GCT cells. We discovered that PRDM14 and CBFA2T2 were present in several GCT cell lines and cooperate to help tumor cells grow and remain undifferentiated.

## 1. Introduction

Germ cell tumors (GCTs) encompass several diverse subtypes, all of which disproportionately affect pediatric and young adult populations. GCTs can arise in both gonadal and extragonadal locations. Regardless of location, GCTs are classified into two major histological categories; seminomatous (known as pure seminoma, dysgerminoma, or germinoma) and non-seminomatous (NSGCT) (including embryonal carcinoma, yolk sac tumor, choriocarcinoma, teratoma, and mixed GCTs) subtypes [1]. Treatment strategies are dependent on subtype, stage, and tumor marker status, with treatment options including surgery, chemotherapy, and radiotherapy (primarily for CNS GCTs). Fortunately, GCTs have been a treatment success story, with the majority of patients achieving long-term remission [2]. However, despite this therapeutic success, GCT patients can experience significant acute and long-term toxicities from therapy, which are particularly harmful for young, developing patients [3]. To optimize survival while minimizing long-term toxicities, therapies directed at the underlying disease mechanism are needed.

The primordial germ cell (PGC) is generally believed to be the cell of origin of GCTs [4]. PGCs are the precursor cells to sperm and eggs, and must undergo specification to a germline fate, migration into the genital ridge, and differentiation into prospermatogonia or oogonia during early embryonic development [5]. Several studies have supported the idea that GCTs arise from PGCs that fail to migrate and/or differentiate appropriately, with extragonadal GCTs originating from PGCs that fail to reach the genital ridge as they migrate along the midline, and gonadal GCTs arising from appropriately migrated but undifferentiated PGCs [6,7,8]. The developmental trajectory of PGCs is tightly regulated by a network of transcription factors and effector genes. PGCs express pluripotency genes such as *NANOG*, *POU5F1*, and *PRDM14* in order to appropriately specify and allow for epigenetic reprogramming, while repressing somatic differentiation. These genes are eventually downregulated as PGCs commit to sex-specific differentiation [5,9]. As a hallmark of GCTs, the aberrant expression of pluripotency genes supports the notion that GCTs arise from failed PGC differentiation and provides a pluripotent framework to target therapeutically [10,11,12,13].

*PRDM14* (PR/SET Domain 14) is a zinc finger transcription factor expressed in human pluripotent stem cells (hPSCs) and PGCs. In hPSCs, PRDM14 is shown to mediate pluripotency, particularly maintaining naïve pluripotency, via epigenetic regulation and direct interaction with other core pluripotency genes [14,15,16,17,18]. Studies using human PGCs and PGC-like cells have shown that *PRDM14* is expressed in early-to-late-stage PGCs and plays an important role in their specification [9,15,16,18,19]. Outside of the developmental window, PRDM14 expression is repressed in adult tissues. However, aberrant PRDM14 expression with a tumorigenic role is noted in a number of malignancies including breast cancer, non-small cell lung cancer, and gastric cancer. Notably, *PRDM14* has been identified as a susceptibility locus for testicular germ cell tumor (TGCT), and the region containing *PRDM14*, chromosome 8q13, has been noted to be amplified in intracranial GCTs [20]. At the protein level, PRDM14 is expressed in many GCT subtypes, excluding teratomas, which are a type of GCT that is fully differentiated into all three germ layers [12]. These findings suggest that failure to repress PRDM14 in PGCs may contribute to sustained pluripotency and lack of differentiation in GCTs [12].

CBFA2T2 is a transcriptional corepressor belonging to the ETO (MTG) family. In mouse embryonic stem cells (mESCs), Cbfa2t2 (Mtgr1) was determined to bind to the SET domain of Prdm14, and the two were found to co-occupy regulatory regions of genes involved in differentiation. Knockdown of *Cbfa2t2* and *Prdm14* in mESCs showed comparable shifts from a pluripotent to differentiated phenotype, suggesting that Cbfa2t2 facilitates Prdm14’s maintenance of a pluripotent state [21]. A second study evaluating Cbfa2t2 and Prdm14 similarly found that Cbfa2t2 regulates pluripotency and germline specification in Cbfa2t2 −/− mice [22]. Furthermore, this study demonstrated PRDM14 is bound to CBFA2T2 in an embryonal carcinoma cell line, NCCIT, which was used in this study as an early germ cell model [22]. Chromatin immunoprecipitation sequencing (ChIP-seq) in NCCIT cells revealed multiple genes as co-targets of PRDM14 and CBFA2T2, many of which were transcription factors involved in lineage commitment [22].

Previous investigations of PRDM14 and CBFA2T2 in stem cells and early germ cell models were conducted in a developmental context. The role of PRDM14 in GCTs remains largely unknown. To address this knowledge gap, we investigated the expression, localization and interaction between PRDM14 and CBFA2T2 in a panel of GCT cell lines, as well as explored the biological significance of *PRDM14* and *CBFA2T2* expression in GCT cell line via expression depletion using small interfering RNA (siRNA), followed by transcriptomic profiling and interrogating the resulting effect on proliferation and survival. Our findings demonstrated that PRDM14 and CBFA2T2 contribute significantly to pluripotency maintenance and proliferation of the GCT cells, supporting their role in tumorigenesis and as a potential therapeutic vulnerability.

## 2. Materials and Methods

### 2.1. Cell Culture

Cell lines NCCIT and NTERA2 were obtained from the American Type Culture Collection (ATCC). NEC8 was obtained from AcceGen. GCT44 was a gift from Dr. Toshi Shioda. NCCIT and NEC8 cells were cultured in RPMI 1640 supplemented with 10% fetal bovine serum (Denville, Holliston, MA, USA), 1% penicillin-streptomycin (PS, Gibco, Waltham, MA, USA), 1% GlutaMax (Thermo Fisher Scientific, Waltham, MA, USA), and 1% non-essential amino acids (NEAA, Gibco) under standard condition (37 °C and 5% CO_2_). NTERA2 and GCT44 were cultured in DMEM medium supplemented with 10% FBS, 1% PS, 1% GlutaMax, and 1% NEAA under standard conditions.

### 2.2. Quantitative Real-Time PCR

Total RNA was extracted from cells using the RNeasy Kit (QIAGEN, Hilden, Germany) following the manufacturer’s instructions. cDNA was generated with 1 µg total RNA using High-Capacity RNA-to-cDNA Reverse Transcription Kit (Applied Biosystems, Waltham, MA, USA). Quantitative PCR (qPCR) was performed on a QuantStudio 7 Flex Real-Time PCR System using TaqMan Gene Expression Assays (Applied Biosystems, 4331182. Assay IDs included *GAPDH* Hs02786624 g1, *PRDM14* Hs01119056 m1, *CBFA2T2* Assay ID Hs00955785_m1, *NANOG* Hs02387400_g1, *SOX2* Hs04234836_s1, and *POU5F1* Hs04260367_gH.) and TaqMan Fast Advanced Master Mix (Applied Biosystems, Waltham, MA, USA). Relative gene expression of *CBFA2T2* was calculated using the 2^−ΔΔCt^ method, normalized to *GAPDH* and HeLa cells. Log_2_ fold change was calculated using normalization to *GAPDH* and to HeLa cells for *PRDM14* expression in GCT lines, and corresponding scrambled control samples for siRNA experiments.

### 2.3. Western Blotting

Total proteins were extracted using Pierce RIPA buffer (Thermo Fisher Scientific) supplemented with 1× Halt protease inhibitor cocktail (Thermo Fisher Scientific). Samples were quantified using the Pierce BCA assay (Thermo Fisher Scientific) according to the manufacturer’s instructions. Proteins were denatured at 96 °C for 10 min and separated by 4–15% SDS-PAGE and transferred to a PVDF membrane. Primary hybridization used antibodies against PRDM14 (1:1000, Cell Signaling Technology, Danvers, MA, USA, 83527), CBFA2T2 (1:1000, Bethyl Labs, Montgomery, TX, USA, A303-593A), GAPDH (Cell Signaling Technology, 5174). After washing with 1× TBST, secondary hybridization was performed with HRP-conjugated anti-rabbit or anti-mouse antibodies (1:5000, Abcam, Cambridge, UK, AB205718 and AB205719, respectively). Chemiluminescence signal was visualized using Clarity Western ECL Substrate (Bio-Rad, Hercules, CA, USA) and imaged with a ChemiDoc Imaging System (Bio-Rad).

### 2.4. Immunofluorescence Staining

NEC8 and NCCIT cells were seeded on glass coverslips in 6-well plates at 3 × 10^5^ cells per well and cultured overnight. Cells were then rinsed with PBS and fixed with 4% paraformaldehyde for 20 min at room temperature, then rinsed in PBS. Fixed cells were permeabilized with 0.1% Triton X-100 in PBS for 15 min, rinsed with PBS, and blocked in 10% bovine serum albumin (BSA) in PBS for 1 h. Coverslips were incubated with primary antibodies (1:100 in 10% FBS in PBS) against PRDM14 (Thermo Fisher Scientific, MA5-24292), CBFA2T2 (Bethyl Labs, A303-593A), mouse IgG (Abcam, ab280974), or rabbit IgG (Cell Signaling Technology, 3900) in a humidified chamber overnight at 4 °C. After 3 washes with PBS, coverslips were incubated with either Alexa Fluor 488-conjugated AffiniPure donkey anti-mouse IgG (Jackson Immunoresearch Laboratories, West Grove, PA, USA, 715-545-150) or Alexa Fluor 594-conjugated AffiniPure donkey anti-rabbit IgG (Jackson Immunoresearch Laboratories, 711-585-152) for 1 h at room temperature. Nuclei were counterstained with DAPI and mounted onto glass slides using a mounting medium with DAPI (Abcam, ab104139). Images were acquired using a Leica SP8 confocal microscope (Leica Microsystems, Wetzlar, Germany) with a 20× objective. Images were obtained in multiple fields, and three biological replicates were obtained for each cell line.

### 2.5. siRNA Transfection

Three distinct Silencer Select siRNAs (Thermo Fisher Scientific) targeting *PRDM14* (PR1-3) and *CBFA2T2* (CB1-3), and a single scrambled non-targeting control (SC) siRNA were transfected directly into 3 × 10^5^ cells per well of a 6-well plate using Lipofectamine RNAiMAX Transfection Reagent (Thermo Fisher Scientific) and Opti-MEM (Gibco). A final concentration of 10 nM siRNA was used. Transfected cells were incubated for 96 h prior to analysis.

### 2.6. Co-Immunoprecipitation

Co-immunoprecipitation was performed using the Pierce Classic Magnetic Co-IP Kit (Thermo Fisher Scientific) according to the manufacturer’s instructions. Briefly, cells grown to 80% confluence in 10 cm dishes were lysed with IP Lysis Buffer (with 0.3 M NaCl) supplemented with Halt protease inhibitor cocktail (Thermo Fisher Scientific). Protein concentration was quantified using the Pierce BCA assay according to the manufacturer’s instructions. Three micrograms of primary antibody for PRDM14 (Cell Signaling Technology, 83527), CBFA2T2 (Bethyl Labs, A303-593A), or anti-rabbit IgG (Cell Signaling Technology, 3900) were incubated with 1000 ug of lysate on a tube rotator overnight at 4 °C. Pre-washed protein A/G magnetic beads were added to the antibody-lysate mixture and incubated on a tube rotator for 2 h at 4 °C. Beads were washed 3 times with IP Buffer and captured by magnet. Bound proteins were eluted in 1× Lane Marker Sample Buffer for 10 min at room temperature. Magnetic beads were removed using a magnetic rack. Cleared eluates were analyzed by SDS-PAGE followed by Western blot using CBFA2T2 or PRDM14 primary antibodies, and Cleanblot secondary antibody (Thermo Fisher Scientific).

### 2.7. EdU Incorporation and Imaging

Cells were seeded on coverslips at 5 × 10^5^ cells per well in 6-well plate format. EdU (5-ethynyl-2’-deoxyuridine) was added to the culture medium at a final concentration of 10 μM and incubated for 3 h to label proliferating cells. Then, cells were fixed with 4% paraformaldehyde for 15 min and permeabilized with 0.1% Triton X-100 in PBS for 20 min at room temperature. EdU staining was performed using the Click-iT EdU imaging kit (Thermo Fisher Scientific) according to the manufacturer’s protocol. Briefly, cells were incubated with the Click-iT reaction cocktail containing Alexa Fluor 488 dye for 30 min at room temperature, protected from light. Nuclei were counterstained and coverslips were mounted onto slides using a mounting medium with DAPI (Abcam, ab104139). Images were acquired using a Leica SP8 confocal microscope with a 20× air objective. Tilescan images were obtained and merged using LASX software (version 3.5.7). Fluorescence signals were recorded for DAPI (nuclei) and EdU (proliferating cells). Image analysis was performed using Imaris software (version 10.0). DAPI and EdU-positive cells were identified using the “Spots” module. The “Colocalize Spots” tool was used to quantify EdU-positive spots colocalized within DAPI-positive cells, and the percentage of EdU-positive cells was calculated by dividing the number of EdU spots colocalized to DAPI spots by the total number of DAPI spots.

Three independent biological replicates were performed for each condition. Statistical analyses were performed using GraphPad Prism (version 11.0.2). Data were analyzed using a repeated-measures one-way ANOVA with Geisser-Greenhouse correction, followed by Dunnett’s multiple comparisons test comparing each siRNA condition to scrambled control. A *p*-value < 0.05 was considered statistically significant.

### 2.8. CellTiter-Glo Luminescent Cell Viability Assay

Approximately 6000 cells per well were seeded into a clear-bottomed, opaque-walled 96-well plate about 96 h following siRNA transfection with SC, PR3, and CB1 siRNAs in NEC8 and NCCIT cells, in two biological replicates. CellTiter-Glo reagent (Promega, Madison, WI, USA) was added to each well in a 1:1 ratio, then the plate was protected from light for all subsequent steps. Plates were mixed for 2 min on an orbital shaker, then incubated at room temperature for 10 min before recording luminescence on a Synergy Microplate Reader (BioTek, Winooski, VT, USA).

### 2.9. RNA-Sequencing

Total RNA was extracted from siRNA-treated cells (SC, PR3, and CB1 siRNAs in NEC8 and NCCIT cells, in two biological replicates) using the RNeasy Kit (QIAGEN) following the manufacturer’s instructions. RNA was quantified using a NanoDrop spectrophotometer (Thermo Fisher Scientific, Waltham, MA, USA). Sequencing was performed on 1000 ng total RNA per sample and library preparation, quality control, and sequencing were performed by GENEWIZ by Azenta Life Sciences. Raw sequencing data was processed using the nf-core [23] rnaseq (v3.18.0) pipeline [24] via Nextflow (v24.10.4) [25], mapping to the Human GRCh38 reference genome with default options. Downstream analysis of Salmon-generated gene counts [26] was performed using R Statistical Software (v4.4.0) [27]. Differential expression analysis between siRNA conditions was performed using DESeq2 [28], with experimental replicate incorporated to remove batch effects. Pathway enrichment analysis was performed via gene set enrichment analysis (GSEA) [29] of the Molecular Signatures Database (MSigDB) “Hallmark” gene sets [30]. Visualizations were created using the ggplot2 [31], ComplexHeatmap [32], and ggvenn [33] packages. 

## 3. Results

### 3.1. PRDM14 Is Highly Expressed in GCT Cell Lines and Interacts with CBFA2T2

**Figure 1:**
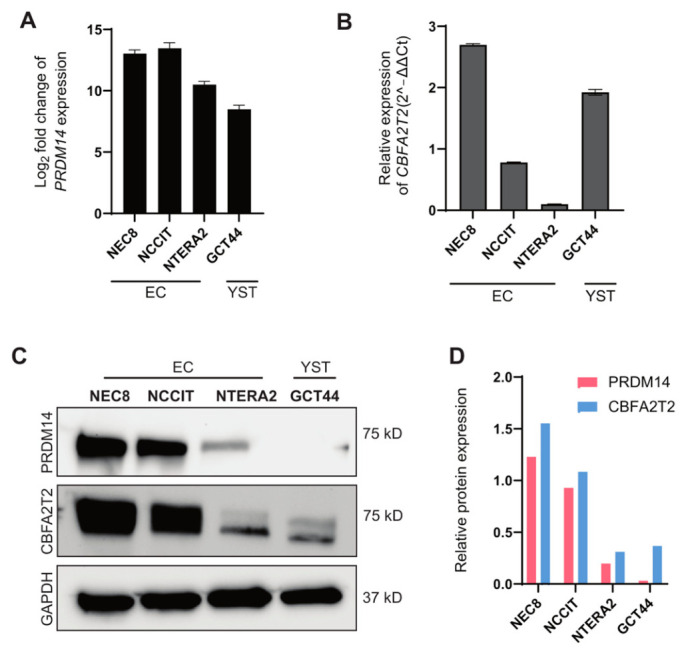
PRDM14 and CBFA2T2 are expressed in germ cell tumor lines. (**A**) Log_2_ fold change in *PRDM14* mRNA expression in a panel of three embryonal carcinoma (EC) and one yolk sac tumor (YST) cell line, calculated normalized to *GAPDH* expression and HeLa cells. mRNA expression data was obtained from qPCR. (**B**) Relative expression (2^−∆∆Ct^) of *CBFA2T2* in NEC8, NCCIT, NTERA2, and GCT44 cells, normalized to GAPDH expression and HeLa cells, obtained from qPCR. qPCR data are shown as mean ± SD from three independent biological replicates. (**C**) Western blot of PRDM14, CBFA2T2, and GAPDH (loading control) in the panel of GCT cells. Each lane contains 50 ug of protein. Blot is representative of three independent biological replicates. The uncropped blots are shown in Supplementary Materials. (**D**) Densitometric quantification of Western blot bands from the representative blot shown. Values represent background-corrected PRDM14 or CBFA2T2/GAPDH ratio.

Our previous investigation demonstrated PRDM14 expression at the protein level in embryonal carcinoma (EC) cell lines and tumor cell samples [12]. However, investigation into PRDM14 and its co-factor CBFA2T2 in the context of GCTs has yet to be explored. To investigate the expression of *PRDM14* and *CBFA2T2* in a series of GCT cell lines, we first assessed mRNA levels of *PRDM14* and *CBFA2T2* across a panel of GCT cell lines using quantitative PCR (qPCR). HeLa cells were used as a reference with low *PRDM14* expression. *PRDM14* is robustly expressed in four GCT cell lines (NEC8, NCCIT, NTERA2, and GCT44), representing two GCT subtypes, EC and yolk sac tumor (YST). These GCT cell lines demonstrated over ten-fold increases in *PRDM14* expression relative to HeLa cells (Figure 1A). *CBFA2T2* was moderately expressed in the panel of GCT lines, with especially high expression in NEC8, an EC line, and GCT44, a YST line (Figure 1B). To further explore expression of PRDM14 and CBFA2T2 in tumor samples, we evaluated mRNA expression correlated with copy number variation from the testicular germ cell cancer TCGA non-seminomatous dataset of 150 patients (Supplementary Figure S1) [34,35,36,37]. Analysis demonstrated frequent gain and amplification of PRDM14, with corresponding increasing mRNA expression. Similarly, CBFA2T2 demonstrates frequent gains and mRNA expression in TGCT samples.

Protein expression of PRDM14 and CBFA2T2 was confirmed by Western blot (WB) analysis (Figure 1C,D). Both proteins were detected in NEC8, NCCIT, NTERA2, and GCT44, with highly varied expression between cell lines. While mRNA expression of *PRDM14* and *CBFA2T2* was moderate to high in GCT44 cells, protein expression in GCT44 was reduced relative to protein expression of PRDM14 and CBFA2T2 in NEC8 and NCCIT cells. NTERA2 cells demonstrated significantly lower mRNA and protein expression of CBFA2T2 compared to the other two EC lines, NEC8 and NCCIT.

**Figure 2:**
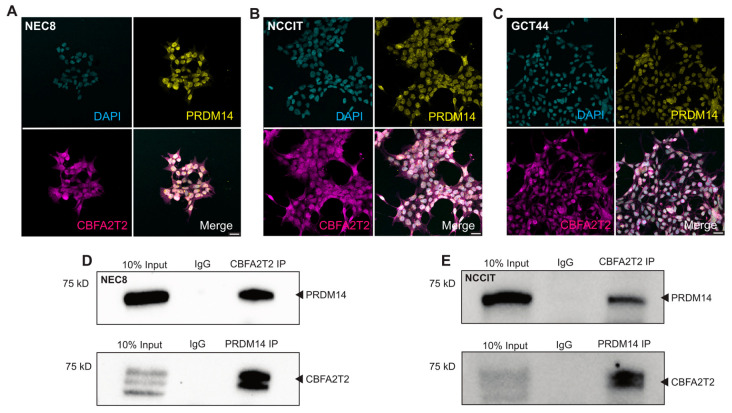
PRDM14 and CBFA2T2 colocalize and interact in GCT cells. (**A**) Representative confocal immunofluorescence (IF) imaging of DAPI (nuclear stain, cyan), PRDM14 (yellow), and CBFA2T2 (magenta) in NEC8 cells grown on coverslips. (**B**) IF imaging of PRDM14 and CBFA2T2 in NCCIT cells grown on coverslips. (**C**) IF imaging of PRDM14 and CBFA2T2 in GCT44 cells grown on coverslips. Scale bars (**A**–**C**) = 25 μm. (**D**) Reciprocal co-immunoprecipitation (Co-IP) in NEC8 shows reciprocal protein interaction of endogenous PRDM14 and CBFA2T2, not due to unspecific IgG binding. Bands shown are representative of three biological replicates. (**E**) Reciprocal co-immunoprecipitation (Co-IP) in NCCIT shows reciprocal protein interaction of endogenous PRDM14 and CBFA2T2. Bands shown are representative of two biological replicates. The uncropped blots are shown in Supplementary Materials.

Spatial distribution of PRDM14 and CBFA2T2 in NEC8, NCCIT, and GCT44 cells was examined by immunofluorescence imaging (Figure 2A–C). PRDM14 exhibited primarily nuclear localization. CBFA2T2 showed more diffuse localization with low expression in the cytoplasm, and enrichment in the nucleus, providing qualitative evidence of their proximity in the nuclei of GCT cells. Next, we sought to determine if PRDM14 and CBFA2T2 interact in GCTs. NEC8 and NCCIT were selected as representatives of EC cell lines for the downstream analysis due to their relatively higher expression of PRDM14 and CBFA2T2 at the protein level. Co-immunoprecipitation (Co-IP) assays using whole cell lysate of high PDRM14 expression GCT cell lines, NEC8 and NCCIT, were performed with anti-PRDM14 or anti-Rabbit IgG antibodies (as negative control) in immunoprecipitation and anti-CBFA2T2 antibody in sequential WB analysis. Reciprocal Co-IP assays were also performed using anti-CBFA2T2 antibody for immunoprecipitation and anti-PRDM14 for WB analysis. As shown in Figure 2D,E, input lanes showed endogenous expression of target proteins. PRDM14 and CBFA2T2 were only detected in the PRDM14- and CBFA2T2-immunoprecipitated samples, but not the IgG control samples. The results confirmed the specific interaction between PRDM14 and CBFA2T2 in NEC8 and NCCIT cells.

### 3.2. Knockdown of PRDM14 or CBFA2T2 Elicits Shared Effects on Pluripotency, Proliferation, and Cancer-Associated Pathways

To explore the biological significance of PRDM14 and CBFA2T2 in GCTs, knockdown experiments using siRNA for *PRDM14* or *CBFA2T2* were performed in NEC8 and NCCIT cell lines. We tested the knockdown efficiency of three siRNAs (PR1-3-KD and CB1-3-KD) per gene in both NEC8 and NCCIT cell lines (Supplementary Figure S4A,B). Among 3 siRNAs for *PRDM14*, PR3-KD had the highest knockdown efficiency (96% in NEC8 and 92% in NCCIT), followed by PR2-KD (90% in NEC8 and 73% in NCCIT) and PR1-KD (81% in NEC8 and 59% in NCCIT) (Supplementary Figure S4A). For *CBFA2T2*, CB2-KD had the highest efficiency (89% knockdown in NEC8 and 81% in NCCIT). CB1-KD and CB3-KD had slightly lower efficiency (75% in NEC8 and 69% in NCCIT for CB1-KD, and 63% in NEC8 and 71% in NCCIT for CB3-KD) (Supplementary Figure S4A). This notable reduction in *PRDM14* and *CBFA2T2* mRNA confirmed respective knockdowns at the transcriptional level. For downstream proliferation and qPCR experiments, siRNAs with the highest knockdown efficiency per gene were chosen.

**Figure 3:**
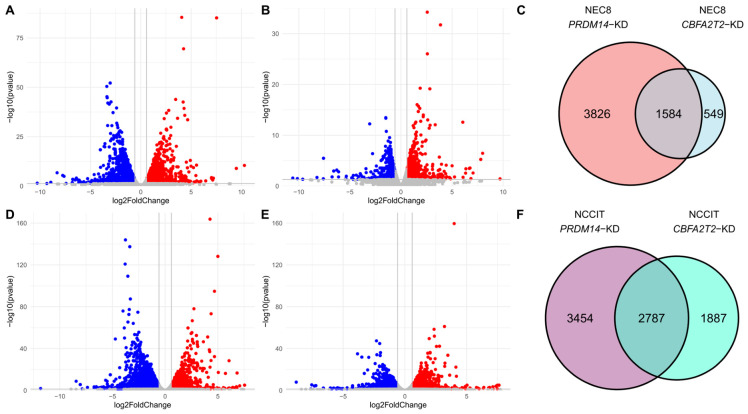
PRDM14 and CBFA2T2 knockdowns affect overall gene expression in GCT cells. (**A**) Volcano plot of significantly (*p* < 0.05) differentially expressed genes in *PRDM14*-KD (PR3) and (**B**) *CBFA2T2*-KD (CB1) in NEC8. (**C**) Venn diagram showing number of significantly differentially expressed genes in *PRDM14*-KD, *CBFA2T2*-KD, and overlap in NEC8. (**D**) Volcano plot of significantly (*p* < 0.05) differentially expressed genes in *PRDM14*-KD and (**E**) *CBFA2T2*-KD in NCCIT. (**F**) Venn diagram showing number of significantly differentially expressed genes in *PRDM14*-KD, *CBFA2T2*-KD, and overlap in NCCIT.

To evaluate the individual and overlapping transcriptional effects of *PRDM14* and *CBFA2T2* knockdown (*PRDM14*-KD and *CBFA2T2*-KD), bulk RNA sequencing (RNA-seq) was performed on duplicate biological replicates 96 h after siRNA transfection. Samples included SC and PR3-KD and CB1-KD, selected as representative siRNAs. These samples were collected from NEC8 and NCCIT cells to verify whether *PRDM14*-KD and *CBFA2T2*-KD had similar effects across multiple GCT cell lines. Gene expression analysis in NEC8 revealed 5410 significantly (*p* < 0.05) differentially expressed genes in *PRDM14*-KD compared to scrambled control (SC), and 2133 significantly differentially expressed genes in *CBFA2T2*-KD versus SC (Supplementary Table S1). Of the differentially expressed genes in each siRNA condition, 1584 genes overlapped (Figure 3A–C). Similarly, knockdown in NCCIT cells revealed 6241 genes significantly differentially expressed as a result of *PRDM14*-KD, and 4674 genes in *CBFA2T2*-KD, with 2787 genes overlapping (Figure 3D–F, Supplementary Table S1).

**Figure 4:**
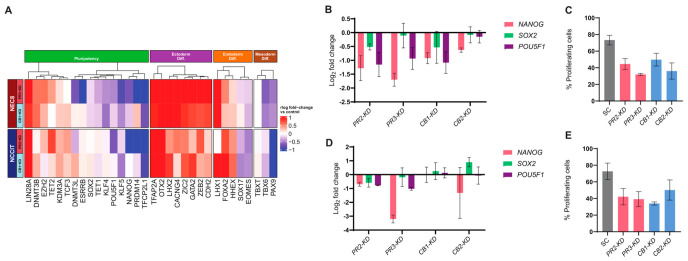
PRDM14 and CBFA2T2 knockdown in GCT cell lines elicit shared effects on pluripotency and proliferation. (**A**) Heatmap of gene expression for established pluripotency and differentiation-associated genes in NEC8 and NCCIT *PRDM14*-KD (PR3-KD) and *CBFA2T2*-KD (CB1-KD). (**B**) Log_2_ fold change in pluripotency gene expression for *NANOG*, *SOX2*, and *POU5F1* in NEC8 cells transfected with *PRDM14* siRNA (PR2-KD and PR3-KD) or *CBFA2T2* siRNA (CB1-KD and CB2-KD). mRNA expression data was obtained from qPCR and normalized to *GAPDH* and NEC8 transfected with scrambled control siRNA. (**C**) Percent proliferating cells relative to total cells in NEC8 treated with scrambled control (SC), *PRDM14*-KD (PR2 and PR3-KD), or *CBFA2T2*-KD (CB1 and CB2-KD). (**D**) Log_2_ fold change in pluripotency gene expression in NCCIT cells. (**E**) Percent proliferating cells in NCCIT. Pluripotency gene expression and proliferation data are shown as mean ± SD from three independent biological replicates.

In an effort to reveal whether *PRDM14*-KD and *CBFA2T2*-KD regulate maintenance of pluripotent markers in GCTs, similar to what has been characterized in stem cells and PGCs [9,15,16,18], RNA-seq data from *PRDM14*-KD and *CBFA2T2*-KD in NEC8 and NCCIT was analyzed for expression of genes related to pluripotency and somatic differentiation (Figure 4A). Notable, major regulators of naïve pluripotency, *KLF5*, *NANOG*, *POU5F1*, and *TFCP2L1* are downregulated in *PRDM14*-KD and *CBFA2T2*-KD. This analysis demonstrates *PRDM14*-KD robustly represses core pluripotency factors, with a similar trend in *CBFA2T2*-KD. Alternatively, *PRDM14*-KD and *CBFA2T2*-KD in EC cells up-regulated differentiation genes. *TFAP2A* and *OTX2* are highly upregulated by repression of *PRDM14* and *CBFA2T2*. Prior work in mouse embryonic stem cells (mESCs) and mPGCs have demonstrated opposing roles, with Prdm14 promoting naïve pluripotency and germ cell specification [38,39]. The downregulated differentiation genes, such as *SOX17*, *EOMES* and *TBXT* are related to specification of the human germline, which may explain the paradoxical downregulation in response to PRDM14 and CBFA2T2 knockdown in the context of GCTs. To validate the decreased expression of pluripotency-associated genes in *PRDM14*-KD and *CBFA2T2*-KD, qPCR was performed with matching RNA samples used for RNA-seq to evaluate mRNA expression of *NANOG*, *POU5F1*, and *SOX2*. *PRDM14*-KD and *CBFA2T2*-KD in NEC8 and NCCIT showed consistent, significant reduction in *NANOG* and *POU5F1* expression, but varied expression of *SOX2*, echoing RNA-seq findings (Figure 4B,D). The variable *SOX2* expression is similar to what has been described in previous studies of PRDM14 knockdown or degradation in hESCs, in which neuronal differentiation is enhanced, including *SOX2* expression [9].

Pluripotent-like states in malignant cells are linked to tumor growth, invasion, and resistance [40,41]. In previous analysis of PRDM14 and other pluripotency factors in the setting of malignancies, dysregulation of pluripotency was also linked to altered proliferation and/or survival [12,42,43,44,45,46]. Therefore, given the evident dysregulation of the pluripotency network in GCT cells lines treated with *PRDM14* or *CBFA2T2* siRNA, we assessed whether this dysregulation would affect proliferation and viability. To test the role of PRDM14/CBFA2T2 on proliferation, a EdU fluorescent imaging assay was performed 96 h after siRNA transfection in NEC8 and NCCIT cells using *PRDM14* (PR2 and PR3-KD), *CBFA2T2* (CB1 or CB2-KD), or SC siRNAs across three independent biological replicates (Figure 4C,E). siRNA treatments demonstrated significant effects on proliferation in both NEC8 (*p* = 0.0282) and NCCIT cells (*p* = 0.0060). Within individual siRNA treatments, a significant (*p* < 0.05) reduction in proliferation was found in PR3-KD vs. SC in NEC8, and in PR2-KD, PR3-KD, and CB1-KD vs. SC in NCCIT cells. A consistent trend toward reduced proliferation was observed across all *PRDM14* and *CBFA2T2*-KDs. However, upon investigation of cell viability following siRNA transfection, using a CellTiter-Glo ATP-based luminescent assay, no effect on cell viability was observed under any siRNA treatment or within either cell type (Supplementary Figure S5). Therefore, *PRMD14*-KD or *CBFA2T2*-KD exerts a cytostatic effect rather than cytotoxic in the context of EC cells.

**Figure 5:**
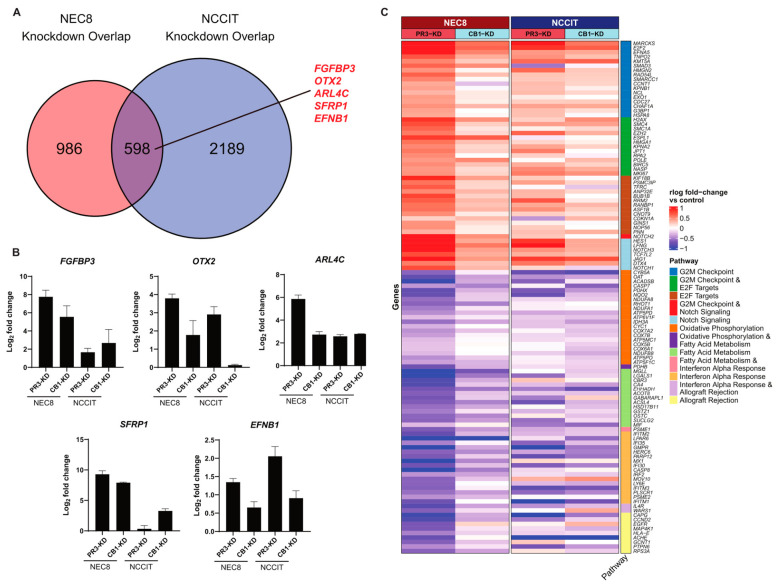
RNA-sequencing analysis of PRDM14 or CBFA2T2 knockdown in GCT lines demonstrates shared effects on top genes and hallmark pathways. (**A**) Overlapping differentially expressed genes in NEC8 and NCCIT from genes that were significantly (*p* < 0.05) differentially expressed in both PRDM14-KD (PR3) and CBFA2T2-KD (CB1) cells. Top five differentially expressed genes by lowest mean *p*-value are highlighted. Data was obtained from RNA sequencing of NEC8 and NCCIT RNA. (**B**) Validation of top 5 differentially expressed genes shared between PRDM14-KD (PR-KD) and CBFA2T2-KD (CB-KD) in NEC8 and NCCIT via qPCR, using matching RNA samples used for RNAseq. (**C**) Heatmap of top altered “Hallmark” pathways shared between PRDM14-KD and CBFA2T2-KD NEC8 and NCCIT cells across one representative replicate. The second replicate is available in Supplementary Figure S6.

To further explore the shared effects of *PRDM14*-KD and *CBFA2T2*-KD, the most significantly differentially expressed genes in *PRDM14*-KD and *CBFA2T2*-KD (*p* < 0.05 in both KD) were compiled into a list for both NEC8 and NCCIT, with 598 differentially expressed genes shared between the two cell lines (Figure 5A). When organizing these 598 genes by the lowest mean *p*-value of *PRDM14*-KD vs. SC and *CBFA2T2*-KD vs. SC in NEC8 and NCCIT, the top five significant genes were *FGFBP3*, *OTX2*, *ARL4C*, *SFRP1*, and *EFNB1*, which were all upregulated in both siRNA conditions and cell lines. These top five differentially expressed genes were validated via qPCR (Figure 5B).

To evaluate the biological significance of the genes significantly affected by both *PRDM14*-KD and *CBFA2T2*-KD, pathway analysis was performed using gene set enrichment analysis (GSEA). In NEC8 cells, gene sets associated with Notch signaling, G2M checkpoint, and E2F targets were significantly positively enriched in both *PRDM14*-KD and *CBFA2T2*-KD conditions compared to the scrambled control. In contrast, gene sets associated with oxidative phosphorylation, fatty acid metabolism, the interferon alpha response, and allograft rejection were significantly negatively enriched (Figure 5C). When this analysis was repeated in NCCIT, a highly similar expression pattern was observed (Figure 5C). To validate the effect of *PRDM14*-KD and *CBFA2T2*-KD on these pathways, qPCR was performed on the samples used for RNAseq, measuring expression of a representative two genes from the top five enriched pathways (Supplementary Figure S7). The directionality of enrichment as determined by qPCR was largely concordant with RNAseq findings, with 18 of 20 measurements matching. Two measurements (NCCIT PR3-KD for *H2AX* and NCCIT CB-KD for *ACADSB*) showed minor discordance (Supplementary Figure S7). This suggests cooperative regulation of cell cycle and metabolic pathways in GCT cells by PRDM14 and CBFA2T2.

## 4. Discussion

In this study, we provide evidence that PRDM14 and CBFA2T2 are co-expressed and functionally linked in GCT cell lines, where they appear to support maintenance of pluripotency-associated transcriptional programs and cellular proliferation. We show that both proteins are present across embryonal carcinoma (EC) and yolk sac tumor (YST) models, colocalize primarily within the nucleus, and can be co-immunoprecipitated under endogenous conditions. Furthermore, depletion of either PRDM14 or CBFA2T2 results in overlapping transcriptional changes, reduced expression of key pluripotency-associated genes, and decreased proliferative capacity. Collectively, these data are consistent with a model in which PRDM14 and CBFA2T2 cooperate to sustain an undifferentiated and proliferative state in GCT cells.

Our results extend prior developmental studies by establishing the PRDM14–CBFA2T2 interaction as biologically significant in a cancer context. In pluripotent stem cells and PGCs, PRDM14 has been shown to recruit transcriptional corepressors to enforce pluripotency and suppress lineage commitment [21,22]. Here, we provide evidence that a similar mechanism may be co-opted in GCTs. The strong overlap in differentially expressed genes following *PRDM14* or *CBFA2T2* depletion, observed across two independent GCT cell lines, suggests that PRDM14 and CBFA2T2 are functionally linked and co-operate to shape the transcriptional program in this context, rather than being merely associated. Notably, core pluripotency factor POU5F1 (OCT4) has been shown to interact with CBFA2T2-containing complexes and facilitate PRDM14 chromatin occupation, suggesting that PRDM14 may operate within a broader pluripotency regulatory network [22]. Together, these observations raise the possibility that additional pluripotency regulators, including NANOG, POU5F1, and SOX2, may also contribute to the function of PRDM14-CBFA2T2 in human cancers, providing further opportunities for investigation.

Transcriptomic and pathway analyses of PRDM14 and CBFA2T2-depleted GCT cells indicate that disruption of this complex promotes a shift away from a pluripotent state toward differentiation. Notably, genes upregulated following *PRDM14* or *CBFA2T2* knockdown include the top five regulated genes: *OTX2*, *ARL4C*, *FGFBP3*, *SFRP1*, and *EFNB1*. *OTX2* is an early neuroectoderm marker [47], and *FGFBP3* and *EFNB1* both positively regulate embryonic neurodevelopment [48,49]. *ARL4C* is a GTP-binding protein associated with progression and proliferation of some cancers, including lung, colorectal, liver, and gastric cancers, yet acts as a tumor suppressor in ovarian cancer [50]. *ARL4C* remains poorly understood in a cancer context but has also been shown to be expressed at high levels in the adult human brain [51], indicating that its upregulation in *PRDM14*-KD and *CBFA2T2*-KD may be connected to differentiation of GCT cells to a neuronal phenotype. SFRP1 is an established negative regulator WNT signaling, a pathway that must be tightly regulated in PGCs in order to maintain a pluripotent state and prevent premature somatic differentiation [52]. Increased expression of *SFRP1* in *PRDM14*-KD and *CBFA2T2*-KD suggests that the two may attenuate expression of SFRP1 in GCTs, allowing cancer cells to remain undifferentiated. Notably, *SFRP1* is also an established tumor suppressor gene, aligning with the idea that PRDM14 and CBFA2T2-mediated pluripotency may be closely tied to malignancy in GCTs [53]. Interestingly, several of these genes (*ARL4C*, *SFRP1*, *EFNB1*) were found to be bound by both PRDM14 and CBFA2T2 in a ChIP-seq study performed in NCCIT cells [22], suggesting a direct role in co-regulating these targets.

Pathway analysis revealed upregulation of genes involved in the G2M checkpoint, E2F targets, and Notch signaling pathways. Conversely, genes involved in oxidative phosphorylation, fatty acid metabolism, interferon alpha response, and allograft rejection were downregulated in both PRDM14 and CBFA2T2 depleted cells. Notch signaling plays a critical role in neurogenesis by regulating cell fate specification, which echoes the discovery of specific neuronal genes being upregulated in PRDM14 and CBFA2T2 knockdowns [54]. G2M and E2F upregulation may indicate departure from a pluripotent state, marked by cell cycle deceleration and increased checkpoint regulation, which is consistent with knockdown of PRDM14 and CBFA2T2 yielding reduced proliferation but unchanged cell viability. Downregulation of oxidative phosphorylation and fatty acid metabolism associated genes may indicate a metabolic adjustment as cells begin to differentiate and adopt a new identity [55,56]. The observed downregulation of metabolic pathways, including oxidative phosphorylation and fatty acid metabolism, further supports this transition. Metabolic reprogramming is tightly coupled to pluripotency, and disruption of PRDM14-CBFA2T2 appears to shift cells away from a metabolic state optimized for rapid proliferation and self-renewal. Notably, these transcriptional and metabolic changes occur in the absence of significant effects on short-term cell viability, and are accompanied by reduced incorporation of EdU, indicating that loss of PRDM14 or CBFA2T2 elicits a cytostatic effect, slowing progression of the cell cycle without inducing acute cell death.

Our findings also align with broader evidence implicating PRDM14 as an oncogenic regulator across multiple cancer types. PRDM14 is aberrantly expressed or amplified in several malignancies and has been linked to maintenance of stem-like states and poor clinical outcomes [57]. In hematopoietic models, PRDM14 interacts with the related ETO-family member CBFA2T3 to drive leukemogenesis, supporting the concept that PRDM14 engages lineage-specific cofactors to exert its oncogenic functions [58,59,60]. Our data suggest that CBFA2T2 plays an analogous role in GCTs, reinforcing the idea that disruption of PRDM14-ETO family interactions may represent a generalizable therapeutic strategy across PRDM14-driven cancers.

Several limitations of this study should be acknowledged. First, our analyses were conducted in established cell lines, which may not fully recapitulate the heterogeneity of primary GCTs. Future studies incorporating additional GCT subtypes or patient-derived samples will be critical to validate the relevance of the PRDM14-CBFA2T2 axis in clinical disease. Second, while our data support a cooperative role for these proteins in regulating transcription, the precise genomic binding landscape of the complex in GCTs remains to be defined. Chromatin-based approaches such as ChIP-seq or CUT&RUN in additional GCT cell lines would clarify whether PRDM14 and CBFA2T2 co-occupy regulatory regions in tumor cells as observed in NCCIT and developmental systems. Further mechanistic studies, including rescue and epistasis experiments, would elucidate the nature of PRDM14 and CBFA2T2’s interaction by determining whether the two are interdependent, which may have implications for future therapeutic intervention in GCTs. Additionally, gain-of-function experiments would further strengthen our conclusion that PRDM14 and CBFA2T2 promote a stem-like proliferative phenotype in GCTs. Migration, invasion, anchorage-independent growth, and colony formation assays could also add valuable context to the relationship between PRDM14-CBFA2T2 and the hallmarks of cancer. Finally, although our findings demonstrate functional dependence of GCT cells on PRDM14 and CBFA2T2 in vitro, the effect of *PRDM14*-KD or *CBFA2T2*-KD on tumor formation in vivo remains to be investigated in future studies.

**Figure 6:**
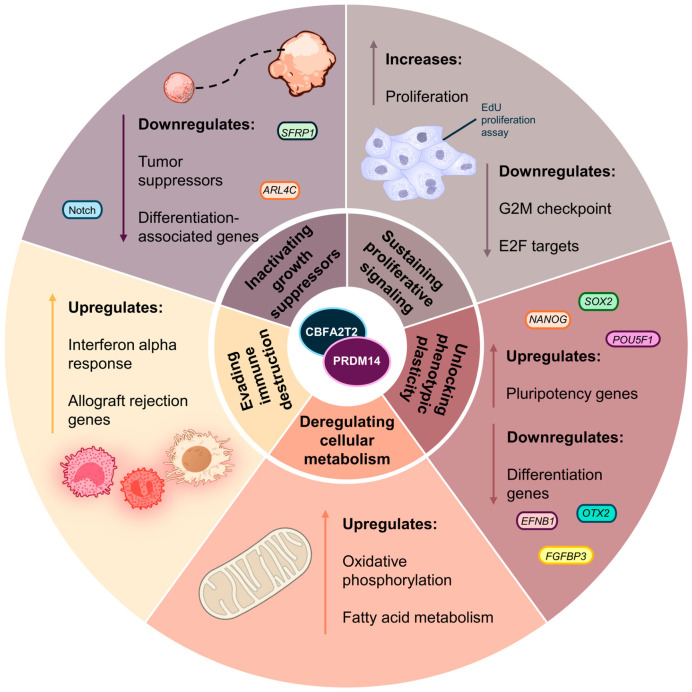
The PRDM14-CBFA2T2 interaction is associated with cancer-relevant genes and pathways in GCTs. Differential gene expression resulting from siRNA knockdown of *PRDM14* and *CBFA2T2* has revealed a network of genes and pathways associated with several hallmarks of cancer [61].

In summary, this study identifies PRDM14 and CBFA2T2 as key contributors to the maintenance of pluripotency and proliferation in GCT cells and provides evidence that their interaction supports the undifferentiated, proliferative state of these cells. These findings support a model in which persistence of germline transcriptional programs contributes to GCT pathogenesis and highlight the PRDM14–CBFA2T2 complex as a potential therapeutic vulnerability (Figure 6). Future efforts aimed at disrupting this interaction or its downstream transcriptional network may offer a targeted strategy to suppress tumor growth while promoting differentiation in GCTs.

## 5. Conclusions

Together, these observations support a model in which PRDM14 engages cell type-specific ETO cofactors (CBFA2T3 in hematopoietic cells, CBFA2T2 in germ cell tumors) to maintain an undifferentiated, proliferative state. By extension, disrupting the PRDM14–ETO cofactor interface may represent a candidate therapeutic vulnerability for GCTs and other *PRDM14*-expressing cancers, and is worth further investigation.

## Data Availability

Bulk RNA-sequencing data for this study are available at the Gene Expression Omnibus (GSE333595).

## Supplementary Materials

The following supporting information can be downloaded at: https://www.mdpi.com/article/10.3390/cancers18132090/s1, Figure S1: PRDM14 and CBFA2T2 are expressed in non-seminomatous GCT (NSGCT) tissue. (A) mRNA expression of PRDM14 in relation to copy number alterations for PRDM14, sourced from TCGA data of 156 non-seminomatous GCT samples from 150 different patients via cBioPortal. (B) mRNA expression of CBFA2T2 in relation to copy number alterations for CBFA2T2; Figure S2: Full membranes of Western blots (WBs) from Figure 1C. (A) Full membrane image of WB probed for PRDM14 (75 kD) in NEC8, NCCIT, NTERA2, and GCT44 cell lysates (50 ug each). (B) Full membrane image of WB probed for CBFA2T2 (75 kD). (C) Full membrane image of WB probed for GAPDH (37 kD); Figure S3: Full membranes of Western blots (WBs) from Figure 2D,E. (A) Full membrane image of WB probed for PRDM14 (75 kD) in NEC8 Co-IP experiment. (B) Full membrane image of WB probed for CBFA2T2 (75 kD) in NEC8 Co-IP. (C) Full membrane image of WB probed for PRDM14 (75 kD) in NCCIT Co-IP. (D) Full membrane image of WB probed for CBFA2T2 (75 kD) in NCCIT Co-IP; Figure S4: PRDM14 and CBFA2T2 knockdown efficiency. (A) Percent knockdown of PRDM14 in NEC8 and NCCIT treated separately with three different siRNAs, as determined by qPCR relative to scrambled control siRNA. Data are shown as mean ± SD from three technical replicates within a single experiment. (B) Percent knockdown of CBFA2T2 in NEC8 and NCCIT treated with three different siRNAs. Data are shown as mean ± SD from three technical replicates within a single experiment; Figure S5: PRDM14 and CBFA2T2 knockdown do not affect cell viability. (A) Cell viability of NEC8 cells treated with PRDM14 (PR3-KD), CBFA2T2 (CB1-KD), scrambled control (SC) siRNA, or treated only with transfection (Tfx) reagent, normalized to untreated cells as determined by Cell-Titer Glo luminescent viability assay. Data are shown as mean ± SD from two independent biological replicates. (B) Cell viability of NCCIT. Data are shown as mean ± SD from two independent biological replicates; Figure S6: Heatmap of top altered hallmark pathways shared between PRDM14-KD and CBFA2T2-KD NEC8 and NCCIT cells across an additional biological replicate to Figure 5; Figure S7: qPCR validation of representative genes within top altered hallmark pathways in PRDM14-KD and CBFA2T2-KD in NEC8 and NCCIT cells. (A) Log_2_ fold change in expression of E2F2 and MARCKS, genes from the G2M Checkpoint pathway, which was upregulated in RNAseq data. Data for qPCR was obtained using matching RNA samples used for RNAseq. (B) Log_2_ fold change in expression of H2AX and ANP32E, genes from the E2F Targets pathway, which was upregulated in RNAseq data. (C) Log_2_ fold change in expression of HES1 and JAG1, genes from the Notch Signaling pathway, which was upregulated in RNAseq data. (D) Log_2_ fold change in expression of MGLL and LGALS1, genes from the Fatty Acid Metabolism pathway, which was downregulated in RNAseq data. (E) Log_2_ fold change in expression of CASP7 and ACADSB, genes from the Oxidative Phosphorylation pathway, which was downregulated in RNAseq data; Table S1: Differential expression analysis of siPRDM14 and siCBFA2T2 treatment versus scrambled control siRNA in NEC8 and NCCIT cell lines. Results from DESeq2 analysis along of each experiment versus their paired control reported. Genes were additionally annotated with PRDM14/CBFA2T2 ChIP-seq binding results in NCCIT, gathered from Tu et al. [22].

## Abbreviations

The following abbreviations are used in this manuscript:

GCT	Germ cell tumor
PGC	Primordial germ cell
mESCs	Mouse embryonic stem cells
siRNA	Small interfering RNA
EC	Embryonal carcinoma
YST	Yolk sac tumor
GSEA	Gene set enrichment analysis
qPCR	Quantitative real-time polymerase chain reaction
WB	Western blot
Co-IP	Co-immunoprecipitation
EdU	5-ethynyl-2’-deoxyuridine
