# Supplementary material for: Interaction Between PRDM14 and CBFA2T2 Supports Pluripotency and Proliferation in Germ Cell Tumors
Source: Cancers (Basel). 2026 Jun 27;18(13):2090. doi: 10.3390/cancers18132090 (PMC13360423; doi:10.3390/cancers18132090)
Supplement: Supplementary file 1 [file cancers-18-02090-s001.zip › Supplementary figures.pdf]

A

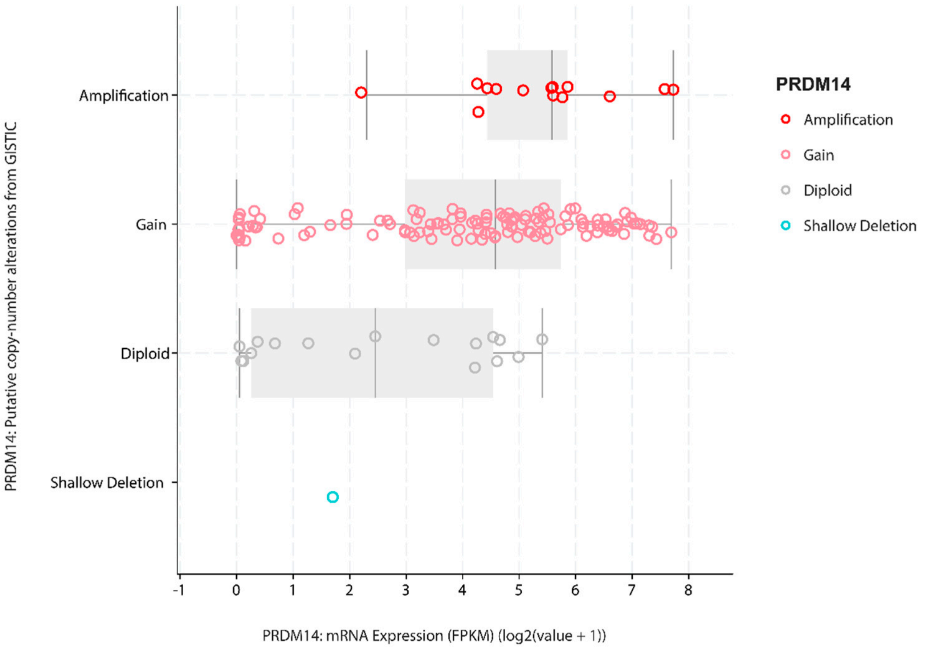

B

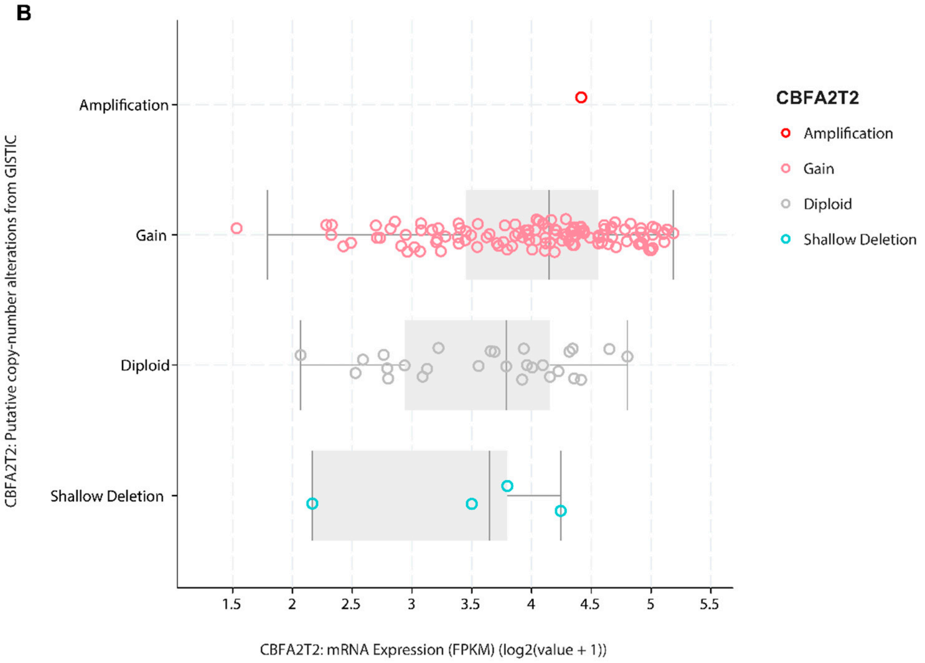

Supplementary Figure S1. *PRDM14 and CBFA2T2 are expressed in nonseminomatous GCT (NSGCT) tissue.* **(A)** mRNA expression of *PRDM14* in relation to copy number alterations for *PRDM14*, sourced from TCGA data of 156 nonseminomatous GCT samples from 150 different patients via cBioPortal. **(B)** mRNA expression of *CBFA2T2* in relation to copy number alterations for *CBFA2T2*.

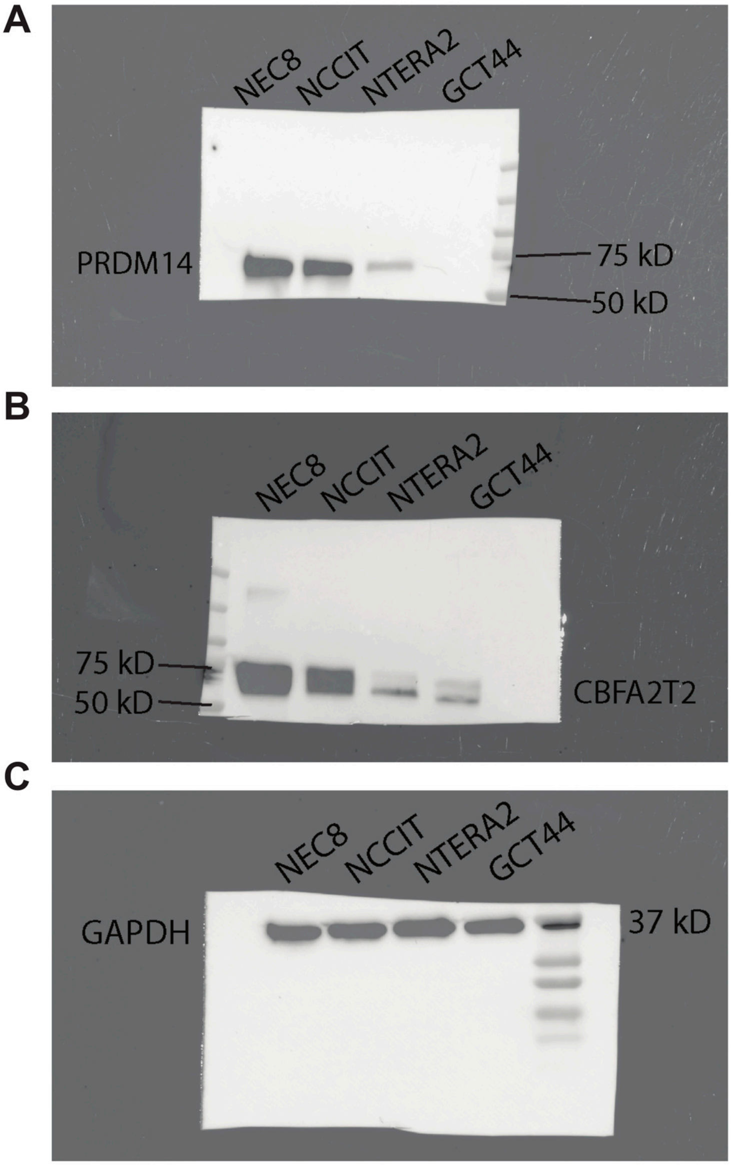

Supplementary Figure S2. *Full membranes of Western Blots (WB) from Fig. 1C.* **(A)** Full membrane image of WB probed for PRDM14 (75 kD) in NEC8, NCCIT, NTERA2, and GCT44 cell lysates (50 ug each). **(B)** Full membrane image of WB probed for CBFA2T2 (75 kD). **(C)** Full membrane image of WB probed for GAPDH (37 kD).

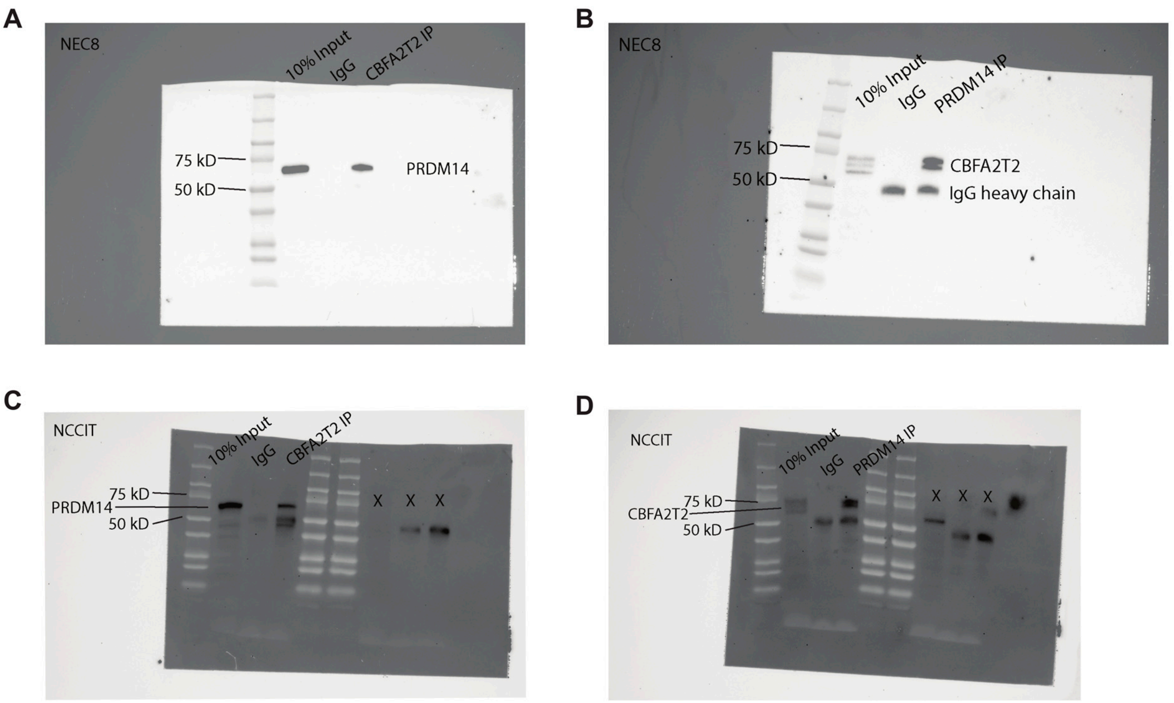

Supplementary Figure S3. *Full membranes of Western Blots (WB) from Fig. 2D and E.* **(A)** Full membrane image of WB probed for PRDM14 (75 kD) in NEC8 Co-IP experiment. **(B)** Full membrane image of WB probed for CBFA2T2 (75 kD) in NEC8 Co-IP. **(C)** Full membrane image of WB probed for PRDM14 (75 kD) in NCCIT Co-IP. **(D)** Full membrane image of WB probed for CBFA2T2 (75 kD) in NCCIT Co-IP.

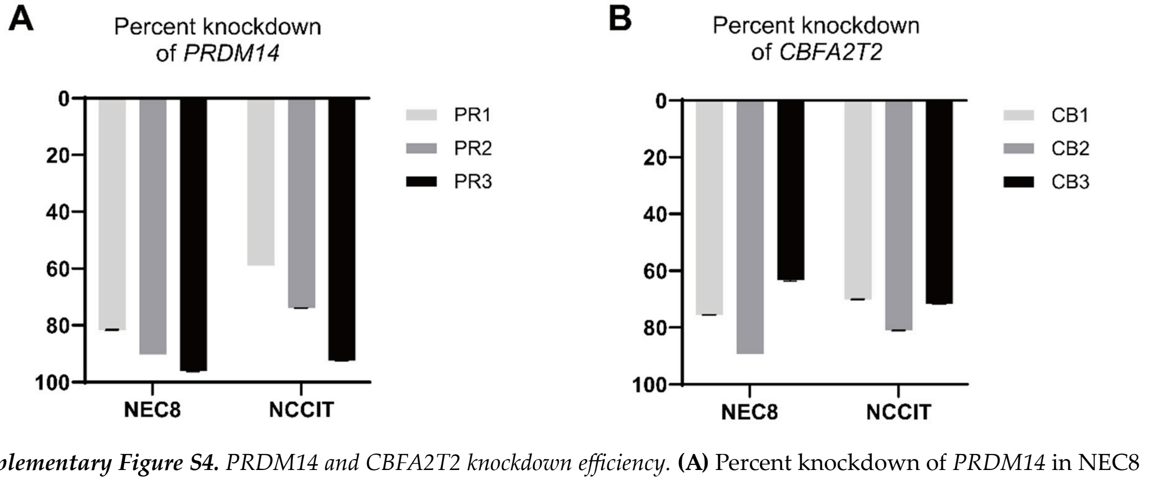

Supplementary Figure S4. *PRDM14 and CBFA2T2 knockdown efficiency.* **(A)** Percent knockdown of *PRDM14* in NEC8 and NCCIT treated separately with three different siRNAs, as determined by qPCR relative to scrambled control siRNA. Data are shown as mean ± SD from three technical replicates within a single experiment. **(B)** Percent knockdown of *CBFA2T2* in NEC8 and NCCIT treated with three different siRNAs. Data are shown as mean ± SD from three technical replicates within a single experiment.

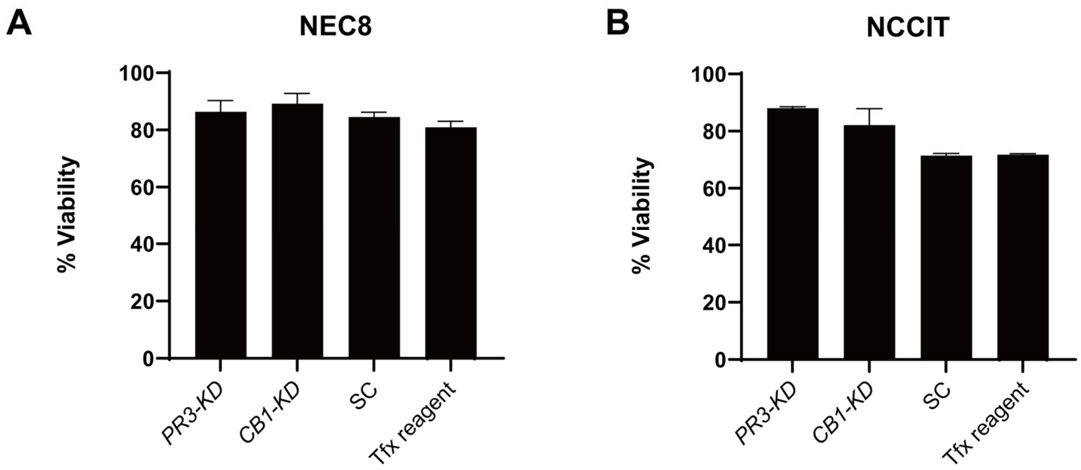

Supplementary Figure S5. *PRDM14 and CBFA2T2 knockdown do not affect cell viability.* **(A)** Cell viability of NEC8 cells treated with *PRDM14* (PR3-KD), *CBFA2T2* (CB1-KD), scrambled control (SC) siRNA, or treated only with transfection (Tfx) reagent, normalized to untreated cells as determined by Cell-Titer Glo luminescent viability assay. Data are shown as mean ± SD from two independent biological replicates. **(B)** Cell viability of NCCIT. Data are shown as mean ± SD from two independent biological replicates.

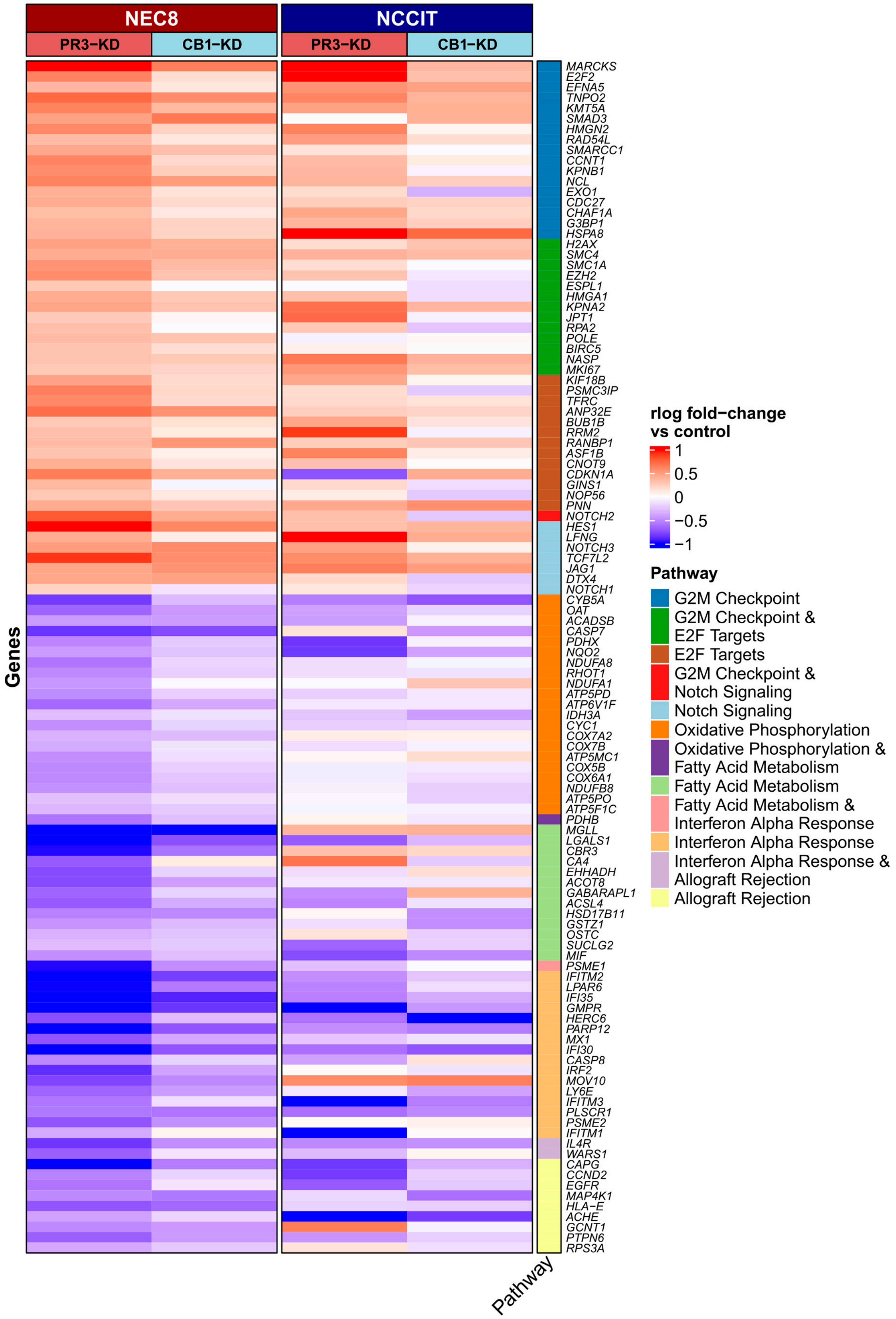

NEC8
NCCIT
PR3−KD
CB1−KD
PR3−KD
CB1−KD
Genes
MARCKS
E2F2
EFNA5
TNPO2
KMT5A
SMAD3
HMGN2
RAD54L
SMARCC1
CCNT1
KPNB1
NCL
EXO1
CDC27
CHAF1A
G3BP1
HSPA8
H2AX
SMC4
SMC1A
EZH2
ESPL1
HMGA1
KPNA2
JPT1
RPA2
POLE
BIRC5
NASP
MKI67
KIF18B
PSMC3IP
TFRC
ANP32E
BUB1B
RRM2
RANBP1
ASF1B
CNOT9
CDKN1A
GINS1
NOP56
PNN
NOTCH2
HES1
LFNG
NOTCH3
TCF7L2
JAG1
DTX4
NOTCH1
CYB5A
OAT
ACADSB
CASP7
PDHX
NQO2
NDUFA8
RHOT1
NDUFA1
ATP5PD
ATP6V1F
IDH3A
CYC1
COX7A2
COX7B
ATP5MC1
COX5B
COX6A1
NDUFB8
ATP5PO
ATP5F1C
PDHB
MGLL
LGALS1
CBR3
CA4
EHHADH
ACOT8
GABARAPL1
ACSL4
HSD17B11
GSTZ1
OSTC
SUCLG2
MIF
PSME1
IFITM2
LPAR6
IFI35
GMPR
HERC6
PARP12
MX1
IFI30
CASP8
IRF2
MOV10
LY6E
IFITM3
PLSCR1
PSME2
IFITM1
IL4R
WARS1
CAPG
CCND2
EGFR
MAP4K1
HLA−E
ACHE
GCNT1
PTPN6
RPS3A
Pathway
rlog fold−change
vs control
1
0.5
0
−0.5
−1
Pathway
G2M Checkpoint
G2M Checkpoint &
E2F Targets
E2F Targets
G2M Checkpoint &
Notch Signaling
Notch Signaling
Oxidative Phosphorylation
Oxidative Phosphorylation &
Fatty Acid Metabolism
Fatty Acid Metabolism
Fatty Acid Metabolism &
Interferon Alpha Response
Interferon Alpha Response
Interferon Alpha Response &
Allograft Rejection
Allograft Rejection

Supplementary Figure S6. Heatmap of top altered hallmark pathways shared between *PRDM14*-KD and *CBFA2T2*-KD NEC8 and NCCIT cells across an additional biological replicate to Figure 5.

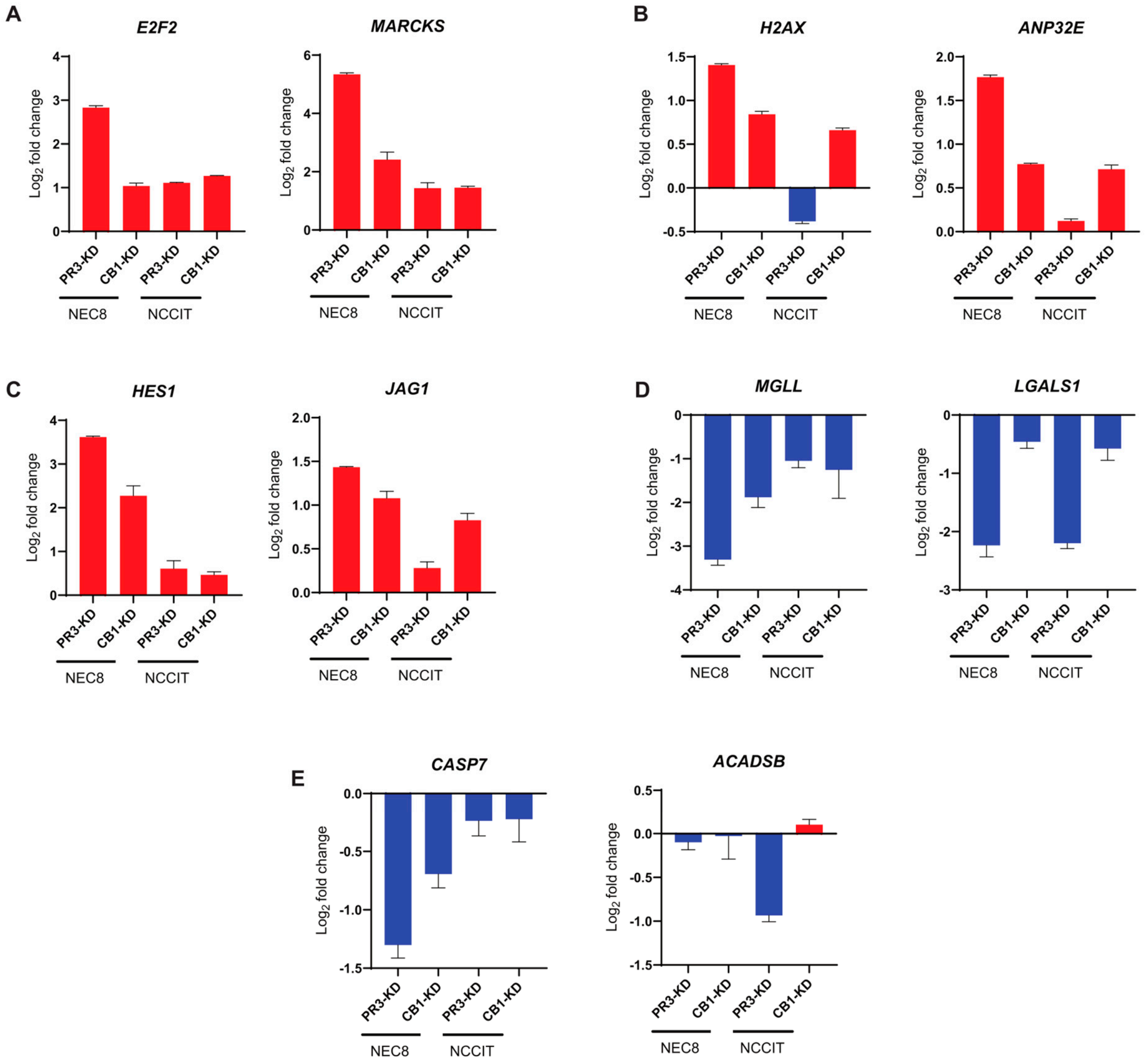

Supplementary Figure S7. *qPCR validation of representative genes within top altered hallmark pathways in PRDM14*-KD *and CBFA2T2-KD in NEC8 and NCCIT cells.* **(A)** Log_2 fold change in expression of *E2F2* and *MARCKS*, genes from the G2M Checkpoint pathway, which was upregulated in RNAseq data. Data for qPCR was obtained using matching RNA samples used for RNAseq. **(B)** Log_2 fold change in expression of *H2AX* and *ANP32E*, genes from the E2F Targets pathway, which was upregulated in RNAseq data. **(C)** Log_2 fold change in expression of *HES1* and *JAG1*, genes from the Notch Signaling pathway, which was upregulated in RNAseq data. **(D)** Log_2 fold change in expression of *MGLL* and *LGALS1*, genes from the Fatty Acid Metabolism pathway, which was downregulated in RNAseq data. **(E)** Log_2 fold change in expression of *CASP7* and *ACADSB*, genes from the Oxidative Phosphorylation pathway, which was downregulated in RNAseq data.
