# Supplementary figures and images for: Interaction Between PRDM14 and CBFA2T2 Supports Pluripotency and Proliferation in Germ Cell Tumors
Source: Cancers (Basel). 2026 Jun 27;18(13):2090. doi: 10.3390/cancers18132090 (PMC13360423; doi:10.3390/cancers18132090)

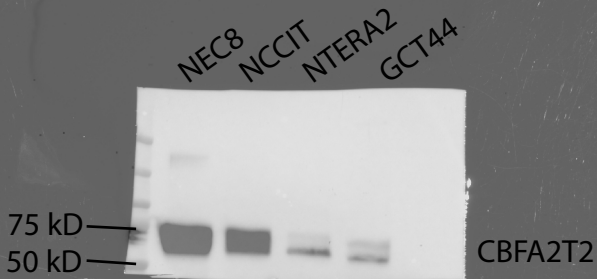

Supplement: Supplementary file 1 [file cancers-18-02090-s001.zip › cancers-4364671 Original Images/Figure 1C CBFA2T2.pdf]

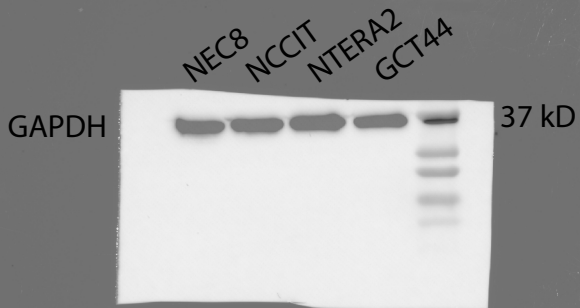

Supplement: Supplementary file 1 [file cancers-18-02090-s001.zip › cancers-4364671 Original Images/Figure 1C GAPDH.pdf]

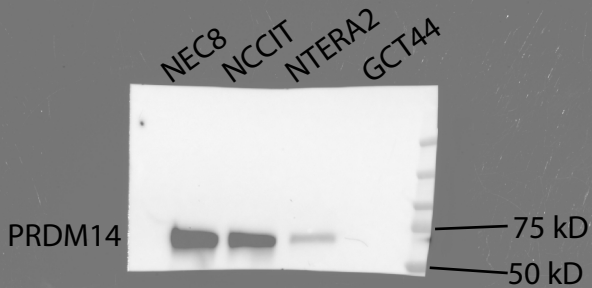

Supplement: Supplementary file 1 [file cancers-18-02090-s001.zip › cancers-4364671 Original Images/Figure 1C PRDM14.pdf]

NEC8

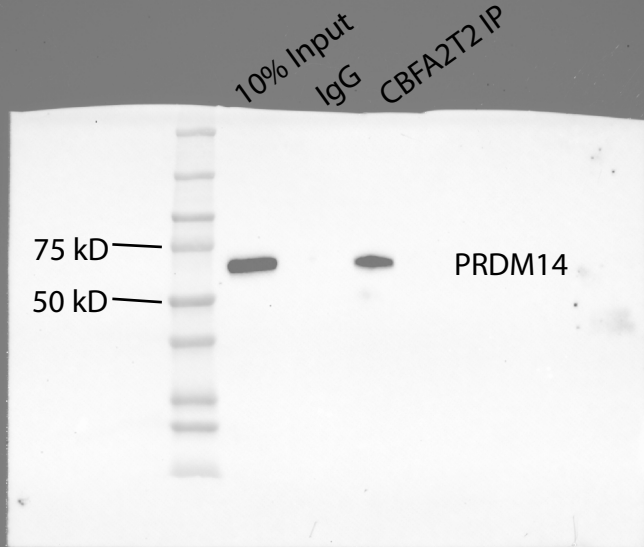

Supplement: Supplementary file 1 [file cancers-18-02090-s001.zip › cancers-4364671 Original Images/Figure 2D NEC8 CB IP.pdf]

NEC8

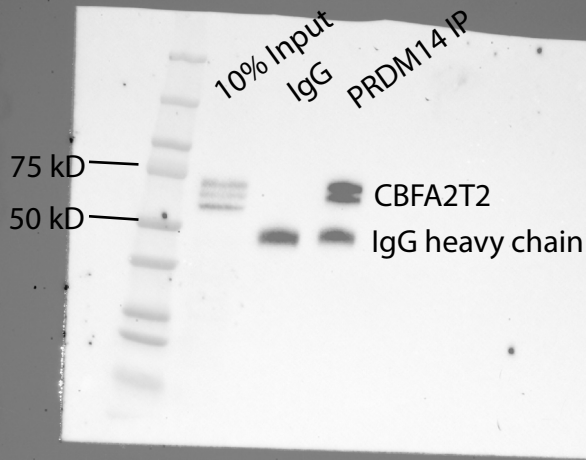

Supplement: Supplementary file 1 [file cancers-18-02090-s001.zip › cancers-4364671 Original Images/Figure 2D NEC8 PR IP.pdf]

NCCIT

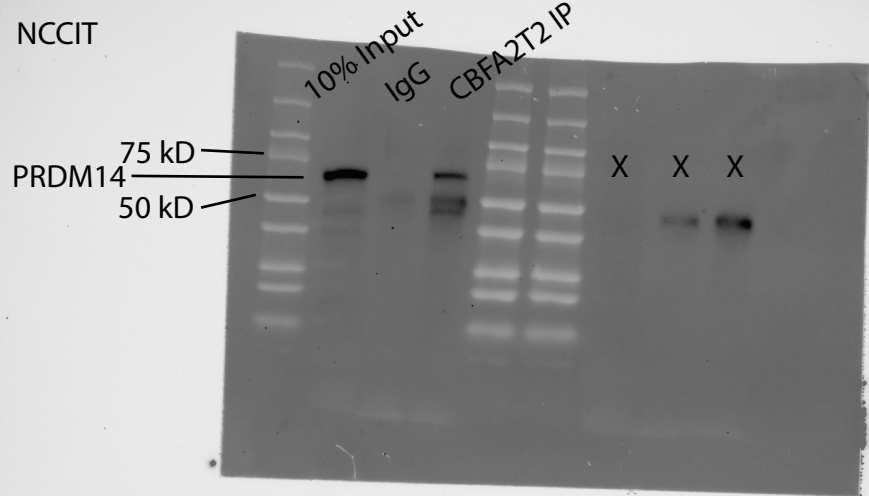

Supplement: Supplementary file 1 [file cancers-18-02090-s001.zip › cancers-4364671 Original Images/Figure 2E NCCIT CB IP.pdf]

NCCIT

75 kD —  
CBFA2T2 —  
50 kD —

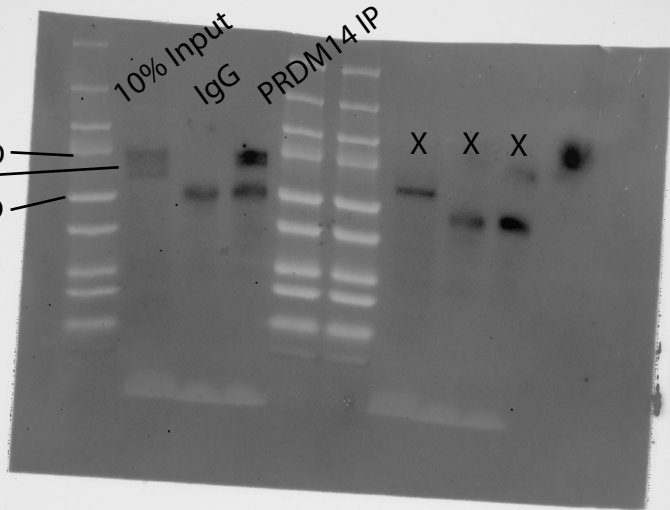

Supplement: Supplementary file 1 [file cancers-18-02090-s001.zip › cancers-4364671 Original Images/‌Figure 2E NCCIT PR IP.pdf]
